# A Lower HCC Incidence in Chronic HBV-Infected Patients Recovered from Acute-on-Chronic Liver Failure: A Prospective Cohort Study

**DOI:** 10.1155/2022/5873002

**Published:** 2022-10-27

**Authors:** Jianhua Yin, Xiang Xu, Rui Pu, Haibin Su, Junxue Wang, Wenbin Liu, Jingjing Tong, Jing Chen, Xi Chen, Xiuying Mu, Hongwei Zhang, Xingran Zhai, Xiaoyan Liu, Fei Pang, Yu Wang, Huifen Wang, Guangwen Cao, Jinhua Hu

**Affiliations:** ^1^Department of Epidemiology, Second Military Medical University, Shanghai, China; ^2^Senior Department of Hepatology, The Fifth Medical Center of Chinese PLA General Hospital, Beijing, China; ^3^Laboratory of Translational Medicine, Medical Innovation Research Division of Chinese PLA General Hospital, Beijing, China; ^4^Department of Infectious Diseases, The 2nd Affiliated Hospital, Second Military Medical University, Shanghai, China; ^5^Medical School of Chinese PLA, Beijing, China; ^6^Peking University 302 Clinical Medical School, Beijing, China; ^7^Qilu Hospital of Shandong University (Qingdao), Qingdao, China; ^8^Ministry of Education Key Laboratory on Signaling Regulation and Targeting Therapy of Liver Cancer, Shanghai Key Laboratory of Hepato-Biliary Tumor Biology, Shanghai, China

## Abstract

**Background:**

Activation of chronic hepatitis B virus (HBV) infection is an important cause of acute-on-chronic liver failure (ACLF). However, the effect of HBV-ACLF episode on hepatocellular carcinoma (HCC) occurrence remains largely unknown.

**Methods:**

A total of 769 HBV-ACLF patients and 2114 HBV-related chronic liver disease (HBV-CLD) patients diagnosed between August 1998 and December 2011 were enrolled in this prospective cohort study. Of the HBV-CLD patients, 380 received lifetime antiviral treatment with nucleos(t)ide analogues. Propensity score matching was applied to reduce baseline differences between HBV-ACLF and HBV-CLD cohorts.

**Results:**

The survival rate of HBV-ACLF patients was 53.6%, 50.3%, 47.8%, and 46.2% at 90-day, 1-year, 5-year, and 10-year, respectively. The cumulative incidence of HCC was lower in HBV-ACLF cohort with 369 eligible patients survived for >90 days than in HBV-CLD cohort with the 380 patients (5.77/1,000 vs. 9.78/1,000 person-years, *p* = 0.0497). HBV-ACLF episode decreased HCC risk regardless of liver cirrhosis, and in patients without family history of HCC. Multivariate Cox analyses indicated that male, increasing age, liver cirrhosis, and platelet count (≤100 × 10^9^/L) increased, whereas HBV-ACLF episode decreased, HCC risk independently. In the propensity score-matched cohorts, HBV-ACLF episode reduced HCC incidence (10.20/1,000 vs. 4.66/1,000 person-years, *p* = 0.0326). The area under curve of nomogram was 0.812 for 3-year HCC probability.

**Conclusions:**

HBV-ACLF episode decreases HCC occurrence in chronic HBV patients. Older age and liver cirrhosis independently increased HCC occurrence. A nomogram-enrolled episode of ACLF reliably predicts the occurrence of HCC.

## 1. Introduction

Acute-on-chronic liver failure (ACLF), a clinical entity distinct from both acute liver failure and decompensated cirrhosis, combines an acute deterioration in liver function in a patient with pre-existing chronic liver disease and hepatic/extrahepatic organ failures, is often associated with a high short-term fatality [1–3]. The pre-existing chronic liver diseases are inflammation, liver fibrosis, and cirrhosis caused by hepatitis virus infection, heavy alcohol consumption, autoimmunity, nonalcoholic steatohepatitis, and genetic diseases [1, 4]. Inflammation-induced tissue damage depends on the intrinsic capacity of host organs to tolerate the intensity of inflammatory responses. A recent study indicated that ACLF episode alone did not affect the long-term outcomes in patients without previous acute decompensation, but it negatively affected long-termtransplant-free survival in patients with previous acute decompensation [5]. Hepatitis B virus (HBV) infection is a pre-existing condition for ACLF. HBV-related ACLF (HBV-ACLF) is prevalent in HBV-endemic countries like China [6]. Reactivation of HBV replication, often triggered by immune depression, withdrawal of nucleos(t)ide analogue (NA), and systemic inflammation, is the most common hepatic insult of HBV-ACLF [7, 8]. ACLF can be triggered by reactivation of HBV replication in patients with or without cirrhosis. Noncirrhotic ACLF has been included in the definition of ACLF by Asian Pacific Association for the Study of the Liver (APASL) [3, 9]. Chronic liver diseases are the prerequisite not only for the occurrence of ACLF but also for the development of hepatocellular carcinoma (HCC). Antiviral treatment with NA is life-timely applied in HBV-ACLF. In HBV-infected patients with antiviral treatment, the 8-year cumulative HCC rate was 4.63% for patients without cirrhosis and 18.87% in patients with cirrhosis [10]. However, HCC and long-term liver death in HBV-ACLF patients with life-time antiviral treatments remain largely unknown. The short-term mortality is the critical outcomes of ACLF, whereas for over half of them would survive this acute episode and suffer other liver-related events including HCC. A small-scale retrospective study indicated that the 5-year HCC incidence rate was higher in HBV patients with decompensated cirrhosis than in patients with HBV-ACLF [11]. In this prospective cohort study, we evaluated HCC occurrence and long-term liver death and their risk factors in HBV-infected patients with an episode of an acute hepatic insult of ACLF.

## 2. Materials and Methods

### 2.1. Study Cohorts

#### 2.1.1. HBV-ACLF Cohort

A total of 1261 consecutive HBV-related liver failure patients admitted to the Fifth Medical Center of Chinese PLA General Hospital, Beijing, China, from September 2006 to December 2011 were enrolled in this cohort. Baseline data were obtained when patients were enrolled in the cohort. These participants were initially diagnosed according to the criteria of Chinese Medical Association [12]. In 2014, the eligible patients were re-identified according to the criteria of the APASL ACLF Research Consortium (AARC) [13]. APASL-AARC-ACLF was an acute hepatic insult manifesting as jaundice (serum bilirubin ≥5 mg/dl [85 micromol/L] and coagulopathy (international normalized ratio [INR] ≥1.5 or prothrombin activity <40%) complicated within 4 weeks by clinical ascites and/or encephalopathy in a patient with previously diagnosed or undiagnosed chronic liver disease/cirrhosis. All enrolled patients were seropositive for hepatitis B surface antigen (HBsAg) for >6 months. Chronic hepatitis B (CHB) and compensated or decompensated cirrhosis were diagnosed by laboratory tests, endoscopy, radiology images, and clinical evidence of previous decompensation and liver biopsy. The exclusion criteria were (1) HCC or systemic malignancy; (2) co-existence of any other severe system diseases; (3) younger than 16 or older than 80 years; and (4) pregnancy. To establish HBV-ACLF cohort, we further excluded patients with decompensated cirrhosis and those who received liver transplantation. The model for end-stage liver disease (MELD) score and chronic liver failure-sequential organ failure assessment (CLIF-SOFA) were evaluated as previously described [2, 14]. Our enrolled patients were also evaluated according to the guideline of European Association for the Study of the Liver-Chronic Liver Failure (EASL-CLIF) [15]. All enrolled patients received supportive treatments, including glycyrrhizin, albumin, artificial liver support, and treatment of complications. Hepatic encephalopathy was treated with lactulose and L-ornithine aspartate. Acute kidney injury was managed by fluid replacement, intravenous albumin, or vasoconstrictors. Bacterial infection was empirically treated with *β*-lactam/*β*-lactamase inhibitors, fluoroquinolones, and third-generation cephalosporins. All patients were seropositive for HBV DNA and received lifetime antiviral treatment with lamivudine, adefovir dipivoxil, or entecavir. The definition of alcohol consumption was based on EASL clinical practice guidelines [16]. Liver cirrhosis in patients with HBV-ACLF was diagnosed by two or more experienced clinicians, based on the results of medical history, ultrasound, and pathological diagnosis or other radiologic tests [17–19], to avoid misdiagnosis or missed diagnosis. Detailed clinical data and outcomes for all enrolled patients were collected at admission, during hospitalization and follow-up. If the patient meets the criteria for ACLF after admission, admission date is considered as baseline date.

#### 2.1.2. HBV-Chronic Liver Disease (CLD) Cohort

**Figure 1:**
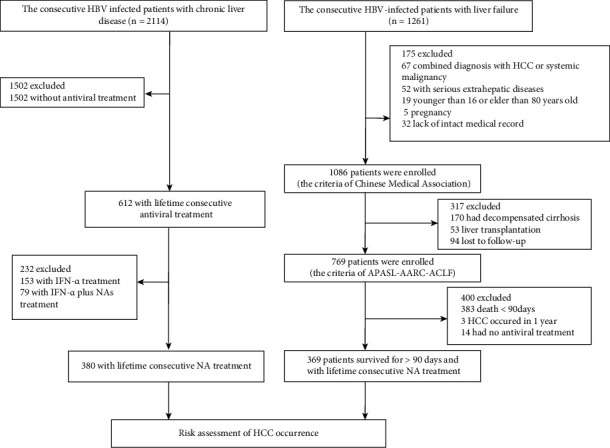
Flowchart of patient enrollments.

A total of 2114 CLD patients admitted to the Department of Infectious Diseases, the Second Affiliated Hospital of Second Military Medical University, Shanghai, China, during August 1998 and December 2007 were enrolled in our former cohort [20]. Baseline data were obtained when patients were enrolled in the cohort. Chronic hepatitis B were diagnosed as previously described [21]. The diagnostic criteria of liver cirrhosis were exactly the same as the HBV-ACLF cohort [17–19]. Of those, 380 HBV-CLD patients were eventually enrolled who receive lifetime consecutive NAs treatment only (Figure 1).

### 2.2. Follow-Up

HBV-ACLF patients were followed-up once a month in the first 6 months after recovered from ACLF and then every 6 months to the end of follow-up. During follow-up, we recorded clinical and laboratory data including demographic data, liver function tests (serum albumin, alanine aminotransferase [ALT], aspartate aminotransferase [AST], total bilirubin [TBil], and INR), sodium, HBV serological markers, HBV DNA levels, and imaging. ALT fluctuation was defined as its level increased above the ULN (40 U/L in males; 35 U/L in females) after ALT conversion. HBV-CLD patients were regularly followed-up every 6 months. HBV DNA reactivation was defined as an increase in serum HBV DNA of 2 log10 IU/mL above nadir or detectable after HBV DNA conversion. In the patients who received a lifetime antiviral treatment, therapeutic regimen of NAs was adjusted as a rescue therapy for those with viral breakthrough. Ultrasonography or enhanced computerized tomography (CT) or magnetic resonance imaging (MRI) and *α*-fetoprotein test were carried out every 6 months to monitor the occurrence of HCC after recovered from ACLF. The endpoint is HCC occurrence. HCC was diagnosed according to the criteria described previously [21]. The follow-up of the two cohorts was finished on August 31, 2019.

### 2.3. Statistical Analysis

Patients who developed HCC or died within 1 year after the enrollment were excluded from the data analysis. Descriptive statistics were expressed as median (inter-quartile range [IQR]) or mean with standard deviation. Mann–Whitney test or *t* test was applied to compare continuous variables as appropriate. Chi-square test or Fisher's exact test was applied to compare categorical data. The Kaplan–Meier method was applied to estimate the cumulative occurrence of HCC. The log-rank test was applied to compare the cumulative occurrence of HCC. Univariate and multivariate Cox proportional hazard regression models were applied to determine variables that predicted HCC occurrence. A nomogram was formulated based on the results of the multivariate Cox proportional hazard regression analyses from the *R* package “rms.” Time-dependent ROC (receiver operating characteristics) curve, which from *R* package “timeROC,” was also applied to evaluate the accuracy of the model/nomogram. Risk score was calculated from the established hazard risk prediction model used by the *R* package “Predict function.” To evaluate the prophylactic effect of an acute hepatic insult of HBV-ACLF on HCC, the propensity score (PS) matching method was applied to balance the significant baseline variables between HBV-ACLF and HBV-CLD cohorts as previously described [20]. The Fine & Gray model was applied for competing risk analysis. Hazard ratios (HR) and 95% confidence intervals (CI) were applied to indicate significant prognostic variable(s). *p* < 0.05 was considered significant. In the HBV-ACLF cohort, the cutoff value of clinical indicators was determined using *X*-tile software. All analyses were two-side and performed using SPSS, version 21 (Armonk, NY), or *R* platform (v 4.0.2).

## 3. Results

### 3.1. Demographic Characteristics of HBV-ACLF Patients

After initial examinations, 1086 ACLF patients who met the criteria of Chinese Medical Association were enrolled. Of those, 317 were excluded (Figure 1). In the remaining 769 HBV-ACLF patients who met the definition of APASL-AARC-ACLF, average age was 42.69 ± 10.94 years and 85.43% was males. The average MELD score and CLIF-SOFA score were 25.99 ± 6.76 and 7 (5–17), respectively. According to the EASL-CLIF criteria, there were 125 patients (16.25%) without organ failure, 470 patients (61.12%) with one organ failure, 135 patients (17.55%) with two organ failures, and 39 patients (5.07%) with three or more organ failures. The clinical characteristics of the 769 HBV-ACLF patients are detailed in Supplementary Table 1.

### 3.2. HCC Occurrence of HBV Patients with or without ACLF

The survival rate of patients with HBV-ACLF was 68.2%, 58.8%, 53.6%, 51.4%, 50.3%, 47.8%, and 46.2% at 28-day, 60-day, 90-day, 180-day, 1-year, 5-year, and 10-year, respectively (Supplementary Figure 1). The baseline and follow-up data of the two cohorts are shown in Table 1. As all of the 369 patients who survived more than 90 days in the HBV-ACLF cohort and the 380 patients in the HBV-CLD cohort received lifetime consecutive NAs treatment, we then compared the cumulative incidences of HCC between the two cohorts. Age, gender, PLT, and AFP did not differ between the cohorts. Compared to the HBV-CLD cohort, the HBV-ACLF cohort had higher rates of liver cirrhosis, alcohol consumption, lower albumin, lower rates of family history of HCC, and higher levels of TBiL, ALT, and AST. In the HBV-ACLF cohort with 369 patients, the 3-, 5-, and 10-year incidence of HCC were 0.9%, 3.5%, and 6.2%, respectively. In the HBV-CLD cohort with 380 patients, the 3-, 5-, and 10-year incidences of HCC were 1.9%, 5.5%, and 9.4% (log-rank test, *p* < 0.05), respectively. The cumulative incidence of HCC was lower in the HBV-ACLF cohort with 369 eligible patients who survived for >90 days than in HBV-CLD cohort with the 380 patients (5.77/1,000 vs. 9.78/1,000 person-years, *p*=0.0497).

**Figure 2:**
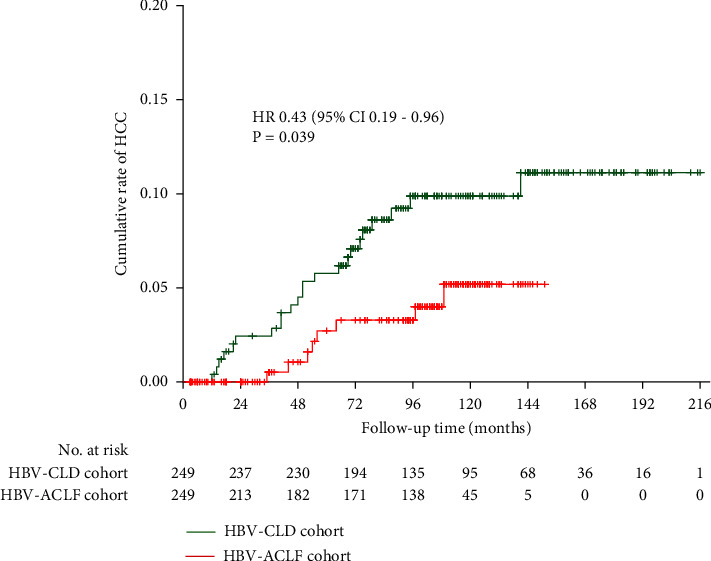
Comparison of HCC occurrence between the HBV-ACLF cohort and HBV-CLD cohorts in matched with propensity score samples.

The PS matching with key baseline characteristics was applied to allow a common background for comparison between HBV-ACLF and HBV-CLD cohorts, resulting in a matched sample size with 249 patients in two cohorts mentioned above. Variables used in the model were age, gender, cirrhosis, and family history of HCC. Age, gender, liver cirrhosis, family history of HCC, HBV DNA, PLT, and AFP did not differ between these two cohorts. It was confirmed that an episode of HBV-ACLF reduced the incidence of HCC (10.20/1,000 vs. 4.66/1,000 person-years, *p* = 0.0326) (Figure 2). To avoid the impact of non-HCC related death which caused censoring on the incidence of HCC, we evaluated the role of ACLF on HCC incidence by a risk competing risk model. The result of a competing risk analysis showed that an episode of HBV-ACLF also reduced the incidence of HCC (HR = 0.404 for adjusted age and gender, 95% CI 0.18–0.92, *p* = 0.03).

### 3.3. Risk Factors of HCC

**Figure 3:**
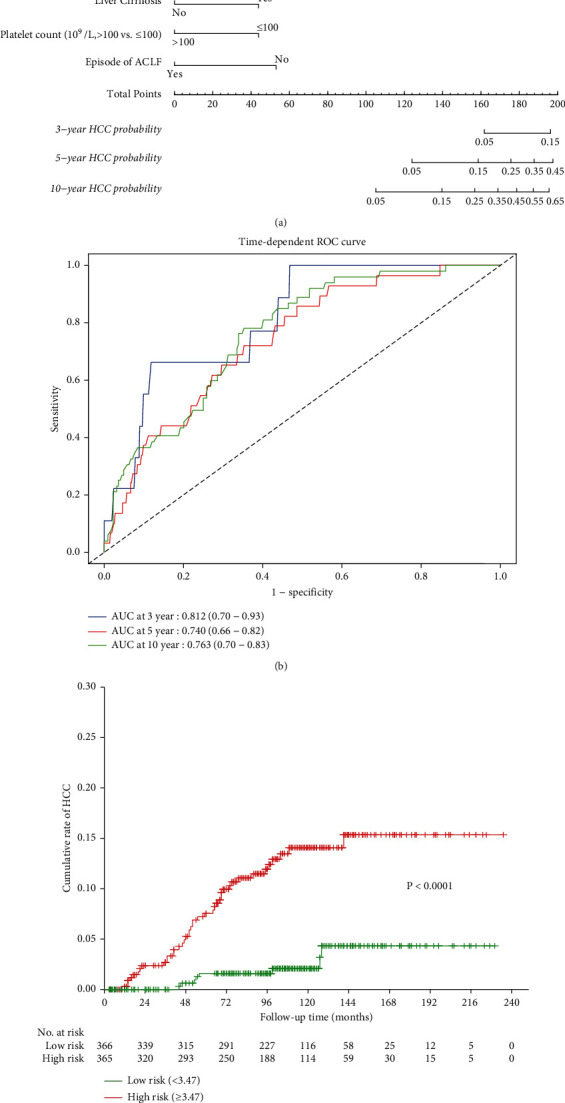
HCC prediction by a nomogram and time-dependent ROC curve.

As liver cirrhosis, family history of HCC, alcohol consumption, and level of HBV DNA were not equally distributed between the HBV-ACLF cohort and the HBV-CLD cohort (Table 1), we stratified the patients in the two cohorts according to the presence of liver cirrhosis, family history of HCC, level of HBV DNA, and alcohol consumption, respectively. In noncirrhotic patients, HCC occurred significantly less in patients with HBV-ACLF episode than in patients with HBV-CLD (Supplementary Figure 2a). The similar result was also observed in cirrhotic patients (Supplementary Figure 2(b)). In patients without family history of HCC, an episode of HBV-ACLF significantly decreased HCC risk (Supplementary Figure 2c); however, this effect was not significant in patients with family history of HCC (Supplementary Figure 2(d)). This effect was not significant in patients with baseline serum HBV DNA of ≥10^4^ copies/mL or HBV DNA of <10^4^ copies/mL, and with/without alcohol consumption (Supplementary Figure 3). We then combined both the cohorts to identify significant factors affected HCC occurrence, adjusted for variables. Sex, increasing age, liver cirrhosis, an episode of ACLF, alcohol consumption, family history of HCC, TBil, albumin, AFP, platelet count, HBV DNA, ALT, and AST were introduced into the univariate Cox model. It was found that male gender, increasing age, the presence of liver cirrhosis, platelet count (≤100 × 10^9^/L), and acute hepatic insult of HBV-ACLF affected HCC risk significantly. Multivariate Cox regression analysis indicated that male gender, increasing age, cirrhosis, and platelet count (≤100 × 10^9^/L) independently predicted HCC occurrence; whereas an episode of HBV-ACLF was an independent protective factor (Table 2). A nomogram composed of these factors was shown in Figure 3(a), and the *C*-index of nomogram was 0.761 (95% CI, 0.73–0.79). The method of time-dependent ROC was applied to evaluate the power of the HCC risk model formulated from multivariate Cox regression analysis in this study. The result demonstrated that the area under curve (AUC) was 0.812 (95% CI, 0.70–0.93) for 3-year HCC probability, 0.740 (95% CI, 0.66–0.82) for 5-year HCC probability, and 0.763 (95% CI, 0.70–0.83) for 10-year HCC probability in these two cohorts (Figure 3(b)). According to the medium of risk score calculated from the established hazard risk prediction model, the whole cohort was classified into high-risk group and low-risk group, the HCC incidence probability in the high-risk group is higher than the low-risk group (*p* < 0.0001) (Figure 3(c)).

### 3.4. Risk Factors of HCC in the HBV-ACLF Cohort

We then evaluated the risks of HCC in the 369 HBV-ACLF patients survived for >90 days. Univariate Cox analysis showed that older age (≥55 years), the presence of cirrhosis, higher TBil, and HBV DNA rebound plus serum ALT fluctuation significantly increased the risk of HCC, whereas platelet count (>100 × 0^9^/L) decreased the risk of HCC. Multivariate Cox analysis indicated that older age (≥55 years), higher TBil, and the presence of liver cirrhosis increased the risk of HCC independently (Table 3).

## 4. Discussion

APASL and EASL/AASLD have a little bit difference in defining ACLF. ACLF can be triggered by reactivation of HBV replication in patients with or without liver cirrhosis. Noncirrhotic ACLF has been included in the definition of ACLF by APASL [22]. One of the main differences between APASL and EASL in the diagnostic criteria of ACLF is that ACLF patients defined by APASL do not include decompensated cirrhosis, but include noncirrhotic patients and compensated cirrhosis patients to represent “chronic,” while EASL-CLIF includes only patients with cirrhosis, whether compensated or decompensated [23]. The discrepancy between the two diagnostic criteria is mainly associated with the diverse etiologies and insults of ACLF.

In this study, ACLF was diagnosed according to the APASL definition, because HBV reactivation, bacterial infection, alcoholism, and superimposed hepatitis virus infection are the precipitating factors of ACLF in China [6, 8, 24]. ACLF precipitated by hepatic insults often identified in Asians is distinct from ACLF precipitated by extrahepatic insults frequently found in Europeans in clinical presentation and prognosis [1, 2, 8, 25]. ACLF fatality increased with increasing organ failure [26]. Patients in whom one organ had failed had a 28-day death of around 20%, which increased to >70% in those in whom three organs had failed [1]. Here, the 28-day death was 31.8%, possibly because of a low percentage of patients at EASL/AASLD ACLF stage *C*.

To determine the contribution of ACLF episode to HCC occurrence, an ACLF-free cohort of 380 eligible HBV-CLD patients was treated with lifetime consecutive NAs treatment as controls. We found, for the first time, that the HBV-ACLF cohort had a lower incidence of HCC, compared to the HBV-CLD cohort. To identify the effect of ACLF episode on HCC after the adjustment for significant variables, it is necessary to combine both cohorts. The reasons to combine the two cohorts were as follows: (1) the enrolled CHB patients were all Chinese Han nationality; (2) the diagnosis, laboratory tests, and clinical treatments followed the same protocols; (3) the diagnostic criteria of liver cirrhosis, an important risk factor and predictor of postoperative prognosis in HCC [27], are the same in the two cohorts; (4) the follow-up procedure and standards were the same; (5) the two cohorts almost had the same number of participants with the same age and gender distributions; and (6) lifetime NAs treatment was equally given to patients in the two cohorts. After combination, an episode of ACLF was proven to be an independent protective factor in HCC. As the distribution of cirrhosis, alcohol consumption, family history of HCC, and level of HBV DNA were not equally distributed between the two cohorts, we stratified study patients according to cirrhosis, alcohol consumption, family history of HCC, and HBV DNA level. Interestingly, the effect of ACLF episode on HCC was not significant in patients with/without alcohol consumption or those with high/low level of HBV DNA, indicating the effect of ACLF episode might be overwhelmed by alcohol consumption and HBV replication background. To further evaluate the prophylactic effect of HBV-ACLF episode on HCC occurrence, PS matching method was applied to balance baseline variables including gender, age, liver cirrhosis, and family history of HCC between HBV-ACLF and HBV-CLD cohorts. The baseline HBV DNA was not balanced because HBV replication in patients of the two cohorts was consistently suppressed since hospitalization. It was found that the incidence of HCC especially long-term incidence of HCC was significantly lower in HBV-ACLF cohort than in HBV-CLD cohort, which was consistent with the result evaluated by competing risk analysis. In the circumstance of potent antiviral therapies, the prognostic significance of the serum HBV DNA level as a biological gradient has substantially diminished [28]. Thus, we concluded that an episode of ACLF significantly decreases the occurrence of HCC in CHB patients.

The mechanism by which an episode of ACLF decreases HCC occurrence remains largely unknown. Compared to the HBV-CLD cohort, HBV-ACLF cohort had higher rates of cirrhosis, TBiL, and lower albumin that often increase HCC risk [20, 29, 30]. Massive liver necrosis may happen in HBV-ACLF patients, thus stimulating the proliferation of hepatic progenitor cells and marked parenchymal replacement by fibrosis and/or necrosis correlated significantly with activation of hepatic progenitors [31]. Patients survived after ACLF often have increasing degrees of cirrhosis, thus leading to an increased risk of HCC theoretically. Surprisingly, our results were quite in contrast to this speculation. ACLF-causing violent inflammation might dis-equilibratehepatocyte-damaging immune activation and tissue-protecting immune suppression, thus contributing to the up-regulation of antitumoral immunity and hepatic damage. We also speculate that some measures after discharge, such as long-term antiviral treatment, prohibition against alcoholic drinks, and stopping taking some liver injury drugs, would impact on the disease prognosis. Further study is needed to clarify this.

HBV DNA rebound plus serum ALT fluctuation was a risk factor of HCC in the HBV-ACLF cohort, indicating that mild but consistent reactivation of HBV increases HCC occurrence. HBV reactivation is mainly caused by NA withdrawal or resistance. Long-term NA treatment decreases HCC development in HBV-infected patients, but cannot eliminate the risk completely [20, 28, 32, 33]. HCC risk is higher when ALT is elevated, but patients with normal ALT are not at low risk for HCC if HBV DNA is detectable [34]. HCC is also related to NA resistance-associated HBV mutations, especially at the position 181 in the reverse transcriptase domain of HBV [35]. Thus, HBV reactivation and ALT fluctuation during follow-up are important in monitoring HCC occurrence in those who recovered from ACLF.

Our study had several limitations. HBV genotypes and mutations were not tested in the HBV-ACLF cohort. These limitations restrict elucidating the role of HBV genotypes and mutations in the mechanisms by which an episode of ACLF decreases HCC occurrence and increases liver death in CHB patients [20, 21, 36]. In addition, more and more evidences showed that the occurrence, development, and outcome of ACLF were mainly related to the interaction between virus (viral load, variation, and evolution) and host (including genetic heterogeneity, age, and gender.) through immune response. The genetic susceptibility and copy number variations were critical for severe hepatitis B flare and have an important role in the pathogenesis of HBV-related ACLF [37–39]. However, one of the limitations of our study is that we did not take into account host genetic factors.

In summary, an episode of ACLF decreases HCC occurrence in CHB patients with or without liver cirrhosis. A nomogram-enrolled episode of ACLF reliably predicts the occurrence of HCC.

## Figures and Tables

**Table 1:** Baseline characteristics and follow-up summary of study patients^a^.

Variables	Entire cohort			Propensity score match cohort^dc^		
	CLD-cohort (*n* = 380)	ACLF-treated cohort (*n* = 369)	*p*	CLD-cohort (*n* = 249)	ACLF-treated cohort (*n* = 249)	*p*
Gender						
Female	68 (17.9)	54 (14.6)	0.227	42 (16.9)	35 (14.1)	0.386
Male	312 (82.1)	315 (85.4)		207 (83.1)	214 (85.9)	
Age (years)^b^	40.5 (34.0–50.0)	42.0 (35.0–49.0)	0.505	42.0 (34.0–51.0)	42.0 (34.5–48.0)	0.130
Liver cirrhosis						
No	278 (73.2)	141 (38.2)	**<0 0.001**	149 (59.8)	141 (56.6)	0.467
Yes	102 (26.8)	228 (61.8)		100 (40.2)	108 (43.4)	
Family history of HCC						
No	348 (91.6)	356 (96.5)	**0.005**	230 (92.4)	236 (94.8)	0.273
Yes	32 (8.4)	13 (3.5)		19 (7.6)	13 (5.2)	
Alcohol consumption						
No	317 (83.4)	257 (69.6)	**<0.001**	211 (84.7)	178 (72.1)	**0.001**
Yes	63 (16.6)	110 (29.8)		38 (15.3)	69 (27.9)	
HBV DNA (log_10_ copies/mL)						
<4.0	58 (15.3)	83 (22.5)	**0.011**	46 (18.5)	63 (25.3)	0.065
≥4.0	322 (84.7)	286 (77.5)		203 (81.5)	186 (74.7)	
HBeAg status						
Negative	190 (50.0)	127 (34.4)	**<0.001**	95 (46.8)	57 (30.6)	**0.001**
Positive	190 (50.0)	242 (65.6)		108 (53.2)	129 (69.4)	
Total bilirubin (*μ*mol/L)^b^	40.0 (21.0–135.0)	261.0 (203.3–349.6)	**<0.001**	47.0 (25.0–142.0)	271.3 (210.4–364.6)	**<0.001**
Albumin (g/L)^b^	36.0 (32.0–40.0)	29.0 (27.0–32.0)	**<0.001**	35.0 (31.0–39.0)	30.0 (27.0–33.0)	**<0.001**
Alanine aminotransferase (U/L)^b^	109.0 (47.0–319.0)	185.0 (91.0–547.0)	**<0.001**	88.0 (43.0–290.3)	214.0 (89.0–606.0)	**<0.001**
Aspartate aminotransferase (U/L)^b^	92.0 (54.0–191.0)	186.0 (116.0–376.0)	**<0.001**	82.5 (54.0–176.0)	199.0 (110.0–391.0)	**<0.001**
Platelet count (10^9^/L)^b^	94.5 (51.8–140.3)	96.4 (66.5–126.0)	0.404	84.0 (47.8–136.3)	105.0 (76.7–131.0)	0.564
*α*-fetoprotein (ng/mL)^b^	17.0 (5.0–100.3)	80.1 (29.5–232.7)	0.543	17.0 (6.0–85.8)	71.0 (28.0–185.2)	0.764
Person-years of follow-up	3575.58	2598.39		2257.42	1718.31	
Follow-up duration (years)	8.8 (6.3–12.3)	8.3 (4.0–9.7)		8.4 (6.2–12.0)	8.2 (3.0–9.6)	
HCC cases	35	15	**0.005**	23	8	**0.005**

*Notes.* ^a^Data are number (%), unless otherwise indicated. Some percentages do not sum up to 100 because of rounding. ^b^Median (IQR). ^c^Variables used in the model were age, gender, cirrhosis, and family history of HCC.

**Table 2:** Cox regression analysis for factors significantly affected the occurrence of HCC in 749 patients enrolled in both of the HBV-ACLF and HBV-CLD cohorts.

Variables	No.(%) of participants (*N* = 749)	Person-years of follow-up	No. of HCC	Incidence per 1000 person-years	Univariate analysis HR (95% CI)	*p*	Multivariate analysis HR (95% CI)	*p*
Gender								
Female	122 (16.3)	1138.5	5	4.39	1		1	
Male	627 (83.7)	5035.5	45	8.94	2.86 (1.11–7.35)	**0.029**	3.08 (1.18–8.04)	**0.021**
Age (IQR, years)	42.0 (34.0–49.5)	6174.0	50	8.10	1.06 (1.03–1.09)	**<0.001**	1.05 (1.18–8.34)	**0.001**
Cirrhosis								
No	419 (55.9)	3744.3	20	5.34	1		1	
Yes	330 (44.1)	2429.7	30	12.35	1.88 (1.06–3.32)	**0.032**	2.08 (1.09–3.97)	**0.027**
Family history of HCC								
No	704 (94.0)	5779.8	45	7.79	1			
Yes	45 (6.0)	394.2	5	12.68	1.66 (0.66–4.19)	0.285		
Alcohol consumption								
No	574 (76.8)	4865.8	37	7.60	1			
Yes	173 (23.2)	1289.7	13	10.08	1.07 (0.56–2.04)	0.840		
HBeAg status								
Negative	317 (42.3)	2530.1	22	8.69	1			
Positive	432 (57.7)	3643.9	28	7.68	1.13 (0.64–1.99)	0.684		
Episode of ACLF								
No	380 (50.7)	3575.6	35	9.79	1		1	
Yes	369 (49.3)	2598.4	15	5.77	0.51 (0.28–0.94)	**0.029**	0.41 (0.22–0.79)	**0.007**
HBV DNA (log_10_ copies/mL)								
<4.0	141 (18.8)	1050.2	10	9.52	1			
≥4.0	608 (81.2)	5124.0	40	7.81	1.00 (0.50–2.02)	0.997		
Alanine aminotransferase (U/L)								
≤40	95 (12.9)	758.3	8	10.55	1			
>40	641 (87.1)	5311.3	41	7.72	0.87 (0.41–1.87)	0.723		
Aspartate aminotransferase (U/L)								
≤37	47 (6.5)	444.7	5	11.24	1			
>37	681 (93.5)	5519.4	42	7.61	0.60 (0.24–1.53)	0.287		
Total bilirubin (*μ*mol/L)								
≤20	88 (12.0)	903.8	4	4.43	1			
>20	648 (88.0)	5176.9	45	8.69	1.37 (0.49–3.85)	0.551		
Albumin (g/L)								
≥35	282 (37.9)	2731.9	19	6.95	1			
<35	462 (62.1)	3411.4	31	9.09	0.99 (0.56–1.78)	0.989		
*α*-fetoprotein (ng/mL)								
≤20	234 (33.9)	2093.0	19	9.08	1			
>20	457 (66.1)	3557.0	27	7.59	0.78 (0.43–1.41)	0.413		
Platelet count (10^9^/L)								
>100	336 (46.0)	2931.8	12	4.09	1		1	
≤100	395 (54.0)	3052.7	37	12.12	2.79 (1.45–5.35)	**0.002**	2.09 (1.04–4.16)	**0.038**

ACLF, acute-on-chronic liver failure; HCC, hepatocellular carcinoma; CLD, chronic liver disease; IQR, inter-quartile range.

**Table 3:** Cox regression analysis for factors affected the occurrence of HCC in the HBV-ACLF cohort.

Variables	No.(%) of participants (*N* = 369)	Person-years of follow-up	No. of HCC	Incidence per 1000 person-years	Univariate analysis HR (95% CI)	*p*	Multivariate analysis HR (95% CI)	*p*
Gender								
Female	54 (14.60)	389.1	2	5.14	1			
Male	315 (85.40)	2209.3	13	5.88	1.53 (0.34–6.98)	0.585		
Age (years)								
<55	320 (86.70)	2253.0	10	4.44	1		1	
≥55	49 (13.30)	345.4	5	14.48	3.43 (1.15–10.26)	**0.027**	4.10 (1.33–12.57)	**0.014**
Cirrhosis								
No	141 (38.20)	1021.9	1	0.98	1		1	
Yes	228 (61.80)	1576.5	14	8.88	9.00 (1.18–68.64)	**0.034**	9.67 (1.25–74.65)	**0.029**
Family history of HCC								
No	356 (96.50)	2512.1	14	5.57	1			
Yes	13 (3.50)	86.3	1	11.59	2.07 (0.27–15.99)	0.486		
Alcohol consumption								
No	257 (70.03)	1846.1	10	5.42	1			
Yes	110 (29.97)	733.8	5	6.81	1.39 (0.45–4.28)	0.563		
HBV DNA (log_10_ copies/mL)								
<4.0	83 (22.5)	582.6	3	5.15	1			
≥4.0	286 (77.5)	2015.8	12	5.95	1.16 (0.33–4.10)	0.824		
Alanine aminotransferase (U/L)								
<101	103 (28.5)	690.4	6	8.69	1			
≥101	258 (71.5)	1845.4	8	4.34	0.53 (0.18–1.54)	0.244		
Aspartate aminotransferase (U/L)								
<170	167 (46.3)	1154.3	8	6.93	1			
≥170	194 (53.7)	1381.6	6	4.34	0.61 (0.21–1.76)	0.361		
Total bilirubin (*μ*mol/L)								
<368.5	282 (78.1)	2027.5	8	3.95	1		1	
>368.5	79 (21.9)	508.3	6	11.80	3.18 (1.06–9.50)	**0.039**	4.06 (1.36–12.08)	**0.012**
Albumin (g/L)								
<28	124 (33.6)	858.8	8	9.32	1			
≥28	245 (66.4)	1739.6	7	4.02	0.43 (0.16–1.20)	0.107		
*α*-fetoprotein (ng/mL)								
<114	218 (60.4)	1475.6	5	3.39	1			
≥114	143 (39.6)	1060.2	9	8.49	3.03 (0.98–9.36)	0.054		
Platelet count (10^9^/L)								
<60	72 (19.5)	483.4	6	12.41	1			
60–100	127 (34.4)	835.5	4	4.79	0.40 (0.11–1.44)	0.161		
>100	170 (46.1)	1279.5	5	3.91	0.29 (0.09–0.95)	**0.039**		
Rebound of HBV DNA and serum ALT fluctuation								
No	338 (91.6)	2339.8	11	4.70	1			
Yes	31 (8.4)	258.6	4	15.47	3.29 (1.04–10.36)	**0.042**		
WBC (×10^9^/L)								
<6.94	207 (57.2)	1436.2	7	4.87	1			
≥6.94	155 (42.8)	1107.9	8	7.22	1.60 (0.58–4.45)	0.365		
Prealbumin (mg/L)								
<26	81 (22.4)	564.4	5	8.86	1			
≥26	280 (77.6)	1971.4	9	4.57	0.55 (0.18–1.66)	0.286		
Sodium (mmol/L)								
<137	182 (50.4)	1269.0	9	7.09	1			
≥137	179 (49.6)	1266.9	5	3.95	0.65 (0.21–1.96)	0.441		
Triglyceride (mmol/L)								
<1.20	204 (56.5)	1420.0	9	6.34	1			
≥1.20	157 (43.5)	1116.4	5	4.48	0.59 (0.20–1.80)	0.358		
Blood ammonia								
<59	170 (47.1)	1166.1	9	7.72	1			
≥59	191 (52.9)	1369.7	5	3.65	0.50 (0.17–1.50)	0.218		
INR								
<1.5	32 (6.1)	138.83	1	7.20	1			
1.5–2.5	300 (83.1)	2132.6	9	4.22	0.64 (0.08–5.08)	0.672		
>2.5	39 (10.8)	264.4	4	15.13	2.67 (0.29–24.81)	0.387		
SBP								
No	283 (76.7)	2029.2	10	4.93	1			
Yes	86 (23.3)	569.2	5	8.78	1.72 (0.58–5.09)	0.325		
Infection (exclude SBP)								
No	335 (90.80)	2407.6	14	5.81	1			
Yes	34 (9.20)	190.8	1	5.24	0.97 (0.13–7.41)	0.977		
Acute kidney injury								
No	309 (83.70)	2207.7	14	6.34	1			
Yes	60 (16.30)	390.7	1	2.56	0.35 (0.05–2.69)	0.314		
Hepatic encephalopathy								
No	322 (87.30)	2238.8	13	5.81	1			
Yes	47 (12.70)	359.6	2	5.56	1.05 (0.25–4.66)	0.953		

ACLF, acute-on-chronic liver failure; HCC, hepatocellular carcinoma; SBP, spontaneous bacterial peritonitis; INR, international normalized ratio.

## Data Availability

The datasets used and analysed during the current study are available from the corresponding author.

## Abbreviations

HBV: Hepatitis B virus
ACLF: Acute-on-chronic liver failure
HCC: Hepatocellular carcinoma
HBV-CLD: HBV-related chronic liver disease
HBV-ACLF: HBV-related ACLF
NA: Nucleos(t)ide analogue
APASL: Asian Pacific association for the study of the liver
AARC: APASL ACLF research consortium
INR: International normalized ratio
HBsAg: Hepatitis B surface antigen
CHB: Chronic hepatitis B
MELD: Model for end-stage liver disease
CLIF-SOFAL: Chronic liver failure-sequential organ failure assessment
EASL-CLIF: European association for the study of the liver-chronic liver failure
ALT: Alanine aminotransferase
AST: Aspartate aminotransferase
WBC: White blood cells.

## Supplementary Materials

Supplementary Materials Supplementary Figure 1. The dynamics of survival rates of the HBV-ACLF patients. Supplementary Figure 2. The effects of liver failure on HCC occurrence in patients with/without liver cirrhosis and family history of HCC. Supplementary Figure 3. The effects of liver failure on HCC occurrence in patients with/without alcohol consumption and the high/low level of HBV DNA. Supplementary Table 1. Basic demographic and clinical characteristics of the 769 HBV-ACLF patients.
