# Supplementary material for: A Lower HCC Incidence in Chronic HBV-Infected Patients Recovered from Acute-on-Chronic Liver Failure: A Prospective Cohort Study
Source: J Oncol. 2022 Oct 27;2022:5873002. doi: 10.1155/2022/5873002 (PMC9633202; doi:10.1155/2022/5873002)
Supplement: Supplementary Materials — Supplementary Figure 1. The dynamics of survival rates of the HBV-ACLF patients. Supplementary Figure 2. The effects of liver failure on HCC occurrence in patients with/without liver cirrhosis and family history of HCC. Supplementary Figure 3. The effects of liver failure on HCC occurrence in patients with/without alcohol consumption and the high/low level of HBV DNA. Supplementary Table 1. Basic demographic and clinical characteristics of the 769 HBV-ACLF patients. [file 5873002.f1.zip › Supplementary Table 1.docx]

**Supplementary Table 1. Basic demographic and clinical characteristics of the 769 HBV-ACLF patients**

| **Characteristics** | **HBV-ACLF** |
| --- | --- |
| **General condition** |  |
| Age (years) | 42.69±10.94 |
| Male | 657 (85.43) |
| Family history of HCC | 22 (2.86) |
| **Number and type of organ failure**^†^ |  |
| **No organ failure** | 125 (16.25) |
| **One organ failure** | 470 (61.12) |
| Liver failure | 434 (56.43) |
| Cerebral failure | 8 (1.04) |
| Coagulation failure | 26 (3.38) |
| Circulation or lung failure | 1 (0.13) |
| Kidney failure | 1 (0.13) |
| **Two organ failures** | 135 (17.55) |
| **Three organ failures or more** | 39 (5.07) |
| **Biochemistry index** |  |
| WBC (×10^9^/L) | 6.8 (1.1- 27.53) |
| PLT (×10^9^/L) | 93 (6-377) |
| Albumin (g/L) | 29.53 ± 4.56 |
| TBiL (µmol/L) | 315.18 ± 126.7 |
| AST (U/L) | 189 (22 - 3494) |
| Creatinine (µmol/L) | 88 (25 - 614) |
| Sodium (mmol/L) | 134.93 ± 7.34 |
| INR | 2.12 ± 0.72 |
| Prothrombin time activity (%) | 32.12 ± 10.01 |
| AFP (ng/ml) | 55 (1 - 4360) |
| **Virology** |  |
| HBV-DNA (log_10_IU/mL) | 4.42 ± 2.54 |
| HBeAg positive | 438 (56.95) |
| **Liver severity score** |  |
| MELD score | 25.99 ± 6.76 |
| CLIF-SOFA score | 7 (5 - 17) |
| SOFA score | 6 (3 - 15) |

**Notes:** Data are expressed as means± SD, median (IQR) or number of patients (%).

^†^ organ failure diagnosed according to the criteria of CLIF-C ACLF.

**Abbreviations:** ACLF, acute-on-chronic liver failure; HBV, hepatitis B virus; HCC, hepatocellular carcinoma; WBC, white blood cells; PLT, blood platelet; TBiL, total bilirubin; AST, Aspartate aminotransferase; INR, international normalized ratio; AFP, alpha fetoprotein; MELD, model for end-stage liver disease; CLIF-SOFA, chronic liver failure-sequential organ failure assessment score; SOFA, sequential organ failure assessment score.
