# Supplementary figures and images for: A Lower HCC Incidence in Chronic HBV-Infected Patients Recovered from Acute-on-Chronic Liver Failure: A Prospective Cohort Study
Source: J Oncol. 2022 Oct 27;2022:5873002. doi: 10.1155/2022/5873002 (PMC9633202; doi:10.1155/2022/5873002)

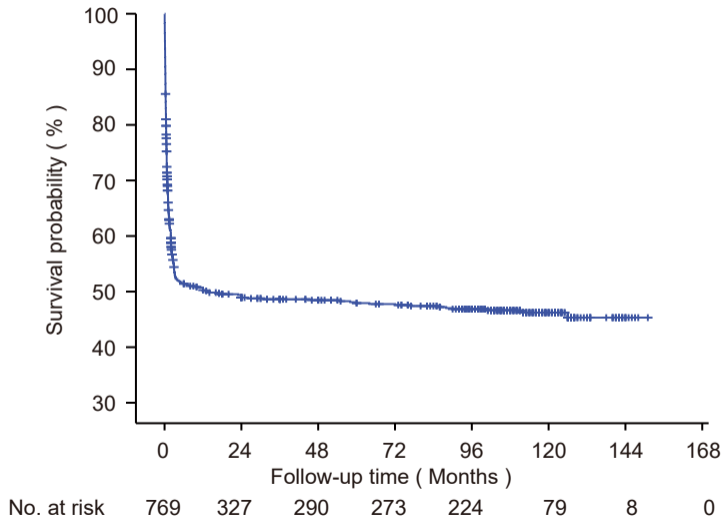

Supplement: Supplementary Materials — Supplementary Figure 1. The dynamics of survival rates of the HBV-ACLF patients. Supplementary Figure 2. The effects of liver failure on HCC occurrence in patients with/without liver cirrhosis and family history of HCC. Supplementary Figure 3. The effects of liver failure on HCC occurrence in patients with/without alcohol consumption and the high/low level of HBV DNA. Supplementary Table 1. Basic demographic and clinical characteristics of the 769 HBV-ACLF patients. [file 5873002.f1.zip › Supplementary Fig.1.pdf]

**A**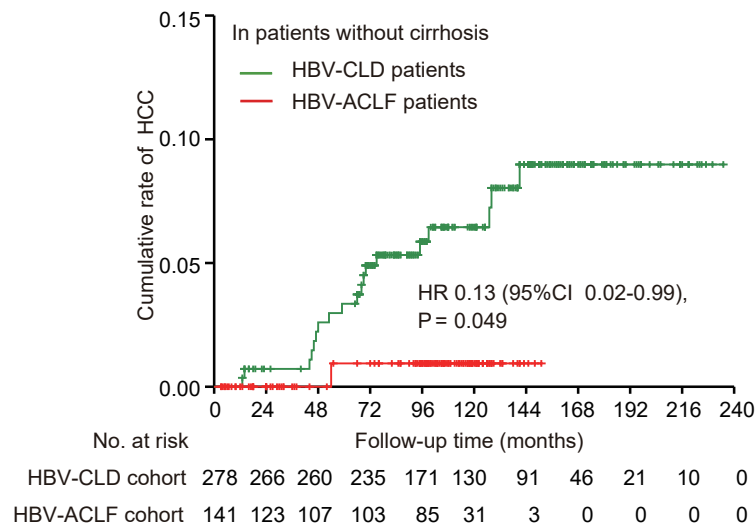**B**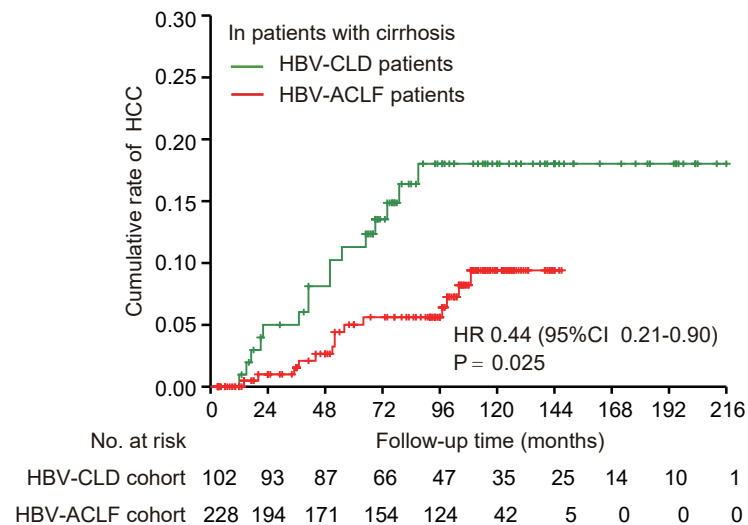**C**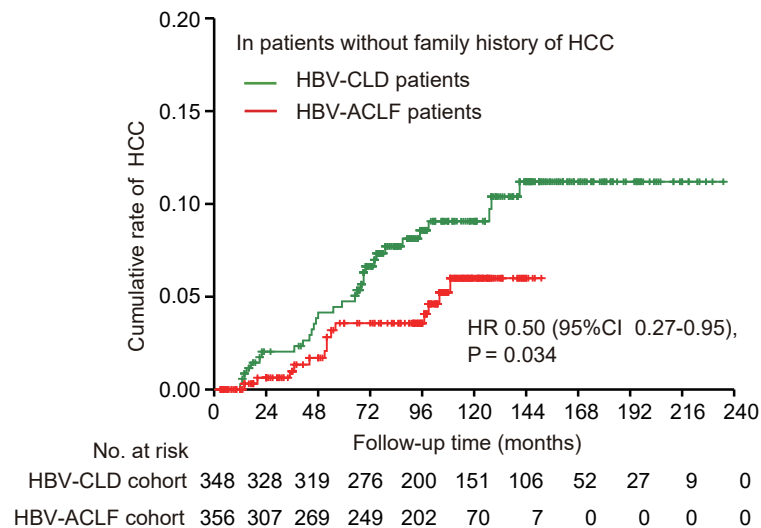**D**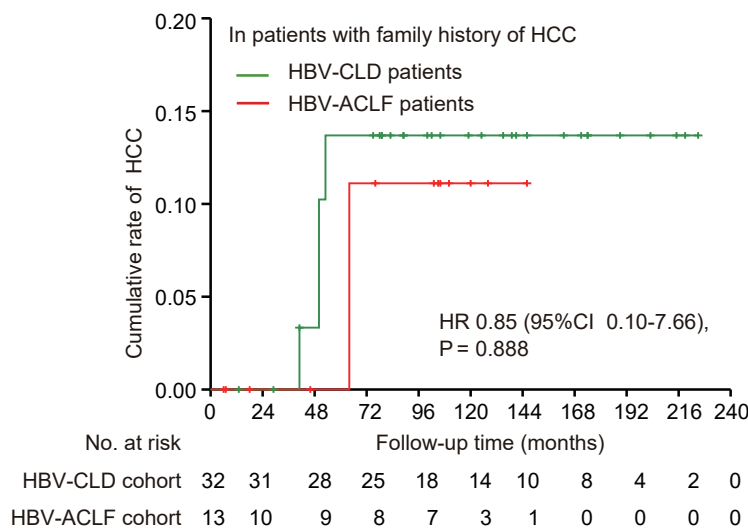

Supplement: Supplementary Materials — Supplementary Figure 1. The dynamics of survival rates of the HBV-ACLF patients. Supplementary Figure 2. The effects of liver failure on HCC occurrence in patients with/without liver cirrhosis and family history of HCC. Supplementary Figure 3. The effects of liver failure on HCC occurrence in patients with/without alcohol consumption and the high/low level of HBV DNA. Supplementary Table 1. Basic demographic and clinical characteristics of the 769 HBV-ACLF patients. [file 5873002.f1.zip › Supplementary Fig.2.pdf]

**A**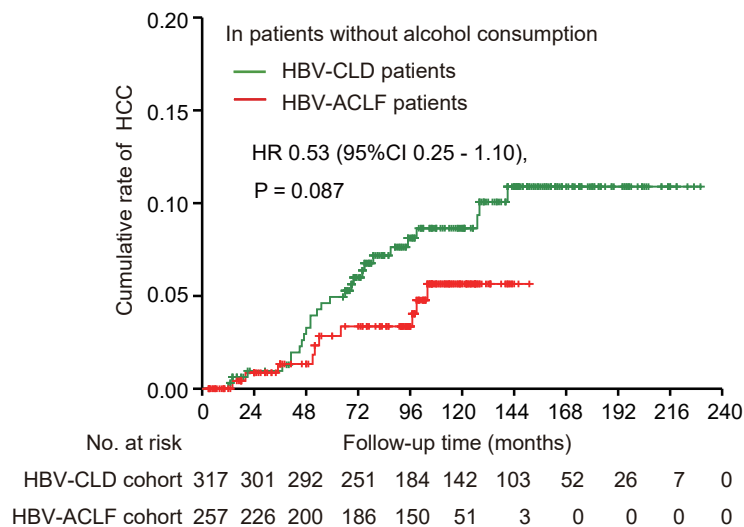**B**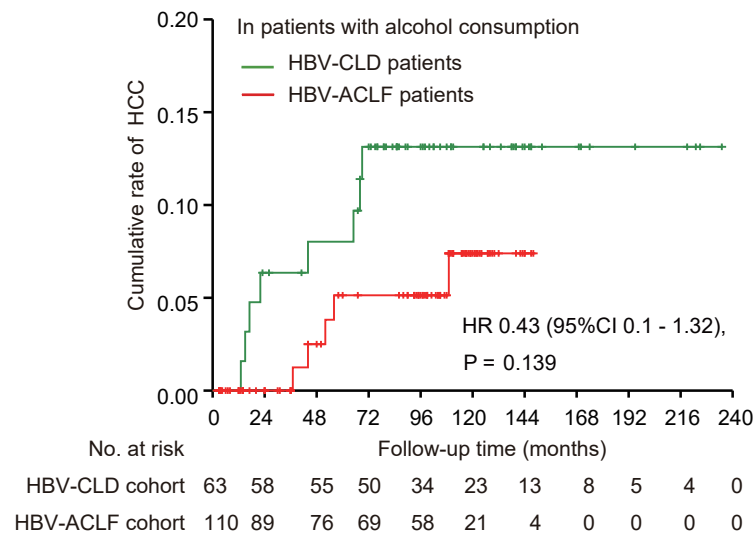**C**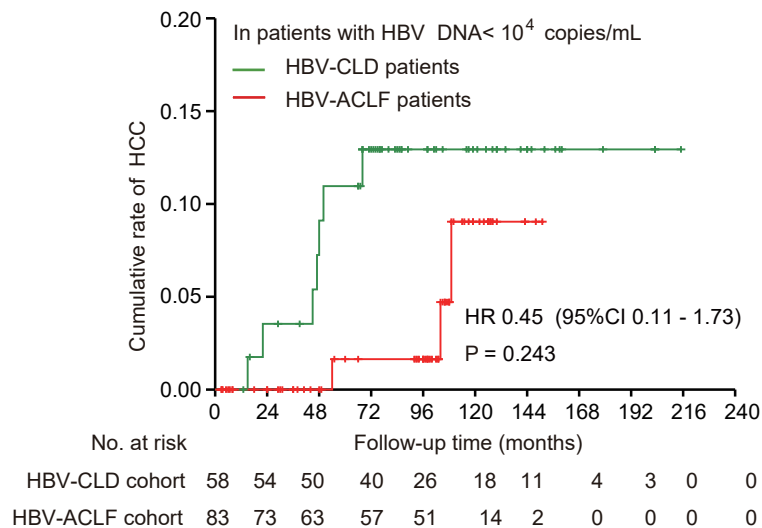**D**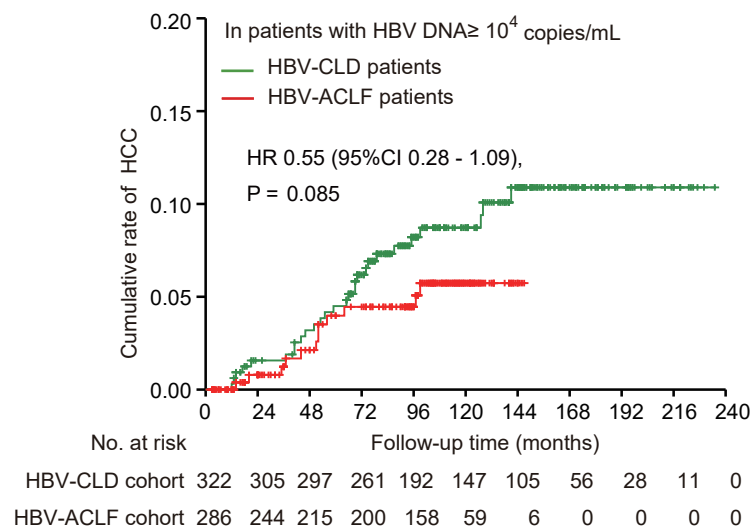

Supplement: Supplementary Materials — Supplementary Figure 1. The dynamics of survival rates of the HBV-ACLF patients. Supplementary Figure 2. The effects of liver failure on HCC occurrence in patients with/without liver cirrhosis and family history of HCC. Supplementary Figure 3. The effects of liver failure on HCC occurrence in patients with/without alcohol consumption and the high/low level of HBV DNA. Supplementary Table 1. Basic demographic and clinical characteristics of the 769 HBV-ACLF patients. [file 5873002.f1.zip › Supplementary Fig.3.pdf]
